# *In Silico* and *In Vitro* Studies on the Protein-Protein Interactions between *Brugia malayi* Immunomodulatory Protein Calreticulin and Human C1q

**DOI:** 10.1371/journal.pone.0106413

**Published:** 2014-09-03

**Authors:** Sunita Yadav, Smita Gupta, Chandrabose Selvaraj, Pawan Kumar Doharey, Anita Verma, Sanjeev Kumar Singh, Jitendra Kumar Saxena

**Affiliations:** 1 Division of Biochemistry, CSIR-Central Drug Research Institute, BS10/1, Sector 10, Jankipuram extension, Lucknow, Uttar Pradesh, India; 2 Computer Aided Drug Design and Molecular Modeling Lab, Department of Bioinformatics, Alagappa University, Karaikudi, Tamilnadu, India; INSERMU1138, France

## Abstract

Filarial parasites modulate effective immune response of their host by releasing a variety of immunomodulatory molecules, which help in the long persistence of the parasite within the host. The present study was aimed to characterize an immunomodulatory protein of *Brugia malayi* and its interaction with the host immune component at the structural and functional level. Our findings showed that *Brugia malayi* Calreticulin (BmCRT) is responsible for the prevention of classical complement pathway activation via its interaction with the first component C1q of the human host. This was confirmed by inhibition of C1q dependent lysis of immunoglobulin-sensitized Red Blood Cells (S-RBCs). This is possibly the first report which predicts CRT-C1q interaction on the structural content of proteins to explain how BmCRT inhibits this pathway. The molecular docking of BmCRT-C1q complex indicated that C1qB chain (IgG/M and CRP binding sites on C1q) played a major role in the interaction with conserved and non-conserved regions of N and P domain of BmCRT. Out of 37 amino acids of BmCRT involved in the interaction, nine amino acids (Pro^126^, Glu^132^, His^147^, Arg^151^, His^153^, Met^154^, Lys^156^, Ala^196^ and Lys^212^) are absent in human CRT. Both ELISA and *in silico* analysis showed the significant role of Ca^+2^ in BmCRT-HuC1q complex formation and deactivation of C1r_2_–C1s_2_. Molecular dynamics studies of BmCRT-HuC1q complex showed a deviation from ∼0.4 nm to ∼1.0 nm. CD analyses indicated that BmCRT is composed of 49.6% α helix, 9.6% β sheet and 43.6% random coil. These findings provided valuable information on the architecture and chemistry of BmCRT-C1q interaction and supported the hypothesis that BmCRT binds with huC1q at their targets (IgG/M, CRP) binding sites. This interaction enables the parasite to interfere with the initial stage of host complement activation, which might be helpful in parasites establishment. These results might be utilized for help in blocking the C1q/CRT interaction and preventing parasite infection.

## Introduction

Lymphatic filariasis, caused by tissue dwelling nematodes: *Wuchereria bancrofti, Brugia malayi and Brugia timori* is considered to be a major obstacle to socioeconomic development in endemic countries (Asia, Africa and Western pacific) and leading cause of permanent and long term disability with morbidity. Over 120 million people have already been affected by the disease. Current control of this disease relies on mass treatment with ivermectin or diethylcarbamazine (lymphatic filariasis) either alone or in combination with albendazole [1]. Existing drugs and control programs have some important limitations with major concern towards the emergence of resistance to ivermectin [2]–[4]. Many parasitic nematodes achieve life spans of years in their host due to effective immune evasion strategies developed by parasites. Most of the processes in immune system occur through an intricate network of protein-protein interactions and any disturbance in this can lead to pathological circumstance. Several excretory and secretory (E/S) products are released by parasites as immunomodulatory factors, which are responsible for modulation or blockage of the effective immune response of the host [5]–[8]. Therefore, identification of these immuno and non-immunomodulatory molecules and their interaction with host immune system at molecular level is necessary not only to understand the host-parasite relationship, but also to develop compounds/drugs for the control of infection.

C1q is the recognition protein of the classical complement pathway and a major connecting link between classical pathway-driven innate immunity and IgG or IgM-mediated acquired immunity [9]. C1q is a 460 kDa protein with N-terminal collagen like stalk (3–81 aa) and C-terminal heterotrimeric globular domain (82–223 aa) [10]. Under normal conditions, approximately 90% of the C1q in circulation exists as C1 complex (C1q-C1r_2_C1s_2_). Binding of the ligands (IgG, IgM, CRP, PTX3) at globular head domain of C1q leads to the auto-activation of C1r, which, in turn, activates C1s [11]–[13]. Crystal structure of globular C1q domain revealed that presence of Ca^+2^ ions stabilized its heterotrimeric structure, which helped in target recognition by C1q [10]. During the activation of C1q-C1r_2_C1s_2_ (C1 complex) cascade in the serum, anaphylotoxic, opsonic, immune stimulating and membrane attacking complex (MAC, lyses of cells) are generated [9], [14], [15]. Although the activation of classical pathway is crucial for host defense, its uncontrolled activation can lead to tissue damage and many diseases [16]. Therefore, current research in biomedicine is important to focus on a detailed structural knowledge of their activation and inhibition.

Calreticulin (CRT), a 46 kDa endoplasmic reticulum (ER) protein which was initially found as a highly pleiotropic calcium binding protein [17], contains globular N, a proline-rich P, and acidic C-terminal domains [17]–[19]. HuCRT shows 54% identity with CRT of *T. cruzi*, *H. contortous, N. americanus, L. donovani and O. volvulus* [20]. Amino acid sequences of both N and P domains of the protein are well conserved among different species, suggesting their important role in the protein function. Mammalian calreticulin is involved in Ca^+2^ cellular buffering [21]–[23], endothelial nitric oxide production [24], molecular chaperon activity [25]–[26] and preventing the aggregation of partially folded proteins and thereby increasing the yield of correctly folded ones [27]–[28]. Human CRT also acts as a “receptor for the C1q collagenous domain” at the surface of phagocytes, since cC1qR which is the receptor for collagenous like stalk of C1q had high homology with HuCRT [29]–[34]. Functional outcome of vertebrate CRT in complement pathway via CRT-C1q complex formation, have been reported only for human C1q [35]–[37]. Extensive literature on the interactions of CRT with C1q has been reported in human emphasizing its important role in the inflammatory processes associated with vascular or atherosclerotic lesions, autoimmune diseases [16], [38]–[40] while in case of parasites (*T. Cruzi, H. contortous, N. americanus*) it is responsible for establishment of infection by preventing host immune response [20], [41]–[43]. However the physico-chemical profile of this protein–protein interaction is still not well understood, while only five to six putative conserved sequences in N and P domain of CRT have been reported on the basis of sequence database and synthetic peptides of these conserve sequences which bind with C1q [36], [44]. It is still not well demonstrated that interaction of C1q with HuCRT involves its GR (globular region), CLF (collagen like fragment), or both domains [29], [35], [36], [45]–[47]. Since last two decades Ghebrehiwet et al., have studied the structure and function of human CRT/cC1qR-C1q interaction and reported that human CRT acts as a C1q receptor which interacts with collagen like stalk of C1q [31], [38]–[40]. Several studies have also suggested binding of CRT to the C1q globular head region [16], [35], [36]. Recently the HuCRT-C1q interaction was analyzed by Paidssai et al, (2011) and they concluded that human CRT shows interaction with both globular head and collagen like stalk of C1q [48]. Their Surface Plasmon Resonance (SPR) kinetic analyses of the binding of full-length C1q to CRT were shown to fit a two state reaction binding model, strongly suggesting a conformational change in C1q that allows CRT to bind C1q, initially to its CLF and then subsequently to its GR. This is in agreements with studies of Steino et al [45]. Mainly ionic interactions are involved in this complex formation [44]. Residues of CRT-C1q interface, which are involved in this interaction, are also not well defined. Efforts to define the three dimensional structure of this complex are thus important and provides much valuable information on the architecture and chemistry of this protein-protein interaction.

No study has been conducted with regards to possible role of filarial CRT, in the modulation of human immune mechanism. Only scant attention has been focused on *N. americanus* hookworm [42]. Thus *Brugia malayi* Calreticulin (BmCRT) is a novel drug target as it shows least homology with human host (56%), *T. cruzi* (38%), and *N. americanus* (58%). The available data allow us to assess its role in the parasite-host relationship in particular, towards the evasion of host immune response and the study of interfaces that mediate these interactions is of prime importance for the understanding its biological function. In the present study we have cloned, expressed, purified BmCRT gene and investigated its potential ability to bind C1q, the initial key component of classical pathway of human complement activation. The interaction was confirmed by haemolytic activity inhibition assay and protein-protein interaction analysis. A 3D model of BmCRT was constructed not only for analyzing whether these two proteins interact, but also to get an insight into the physico-chemical profile and residues (sites) at the protein interface. This is probably the first report to the best of our knowledge which demonstrate CRT-C1q interaction at the structural level of proteins. These findings will be important in understanding the mechanism of host (human)-parasite (*B. malayi*) interactions and may help in reducing the chances of infection.

## Materials and Methods

### 2.1 Preparation of *Brugia malayi* antigens

Adult worm, L3, and Mf of *B. malayi* were isolated from infected *Mastomys coucha* and E/S products of adult worms were collected in RPMI 1640 culture media as reported earlier [49]–[52]. The procedure included incubation of nearly 80 parasites in 1 ml medium for 6 h. The supernatant containing E/S product was collected by centrifugation at 10,000 rpm for 10 min and stored at −20°C. The soluble extract was obtained by centrifuging homogenized 1000–2000 L3 and all other life stages (Adult, Mf) of parasite in 300 µl PBS at 10,000 rpm for 20 min. The studies on animals were approved by the Institutional Animal Ethics Committee (IAEC) of CSIR-CDRI, Government of India.

### 2.2 PCR amplification and cloning of BmCRT gene

According to the sequence information available at NCBI (www.ncbi.nlm.nih.gov) (ID: XP_001896170.1) BmCRT gene was used for designing the specific primers 5′ CATATGCAGCTGTATTTACTGTTAGGACTTG3′(forward)and5′CTCGAGCAGCTCTTCATGTGTTTCATCATC3′ (reverse) for PCR amplification with *NdeI* (CATATG) restriction site at the 5′ end and *XhoI* (CTCGAG) site at the 3′ end. PCR amplification consisted of 30 cycles (30 s at 94°C, 1 min at 57.7°C and 2 min at 72°C), followed by extension cycle (10 min at 72°C) on a PTC 200 PCR system (MJ Research, USA). The amplified BmCRT PCR product was purified and ligated into pGEMT-easy cloning vector. *E. coli* DH5α cells were transformed with the ligated product and grown overnight on agar plates supplemented with 100 µg/ml ampicillin. Correct recombinants were identified by restriction digestion and sequencing. This recombinant plasmid (BmCRT+ pGEMT-easy) was further sub-cloned in PET 28a+ expression vector system using *NdeI* and *XhoI* restriction enzymes. Positive clones were checked for expression of protein by IPTG (1 mM isopropyl-β-thiogalactopyranoside) induction.

### 2.3 Over-expression and purification of BmCRT

BL21 (DE3) pLysS cells containing the recombinant plasmid coding 6xHis-BmCRT were grown at 18°C in LB medium, supplemented with 50 µg/ml kanamycin, shaking at 180 rpm. Culture was induced by addition of 1 mM IPTG for over expression of the gene of interest and grown for an additional 16–18 h at 20°C with shaking and cells were harvested by centrifugation at 10,000 rpm for 3 min. For protein purification, lysis buffer (50 mM Na_2_HPO_4_, 200 mM NaCl, 10 mM Imidazole, pH 7.6) was added to cells pellet containing 1 mM PMSF, 5 mg/ml lysozyme, 1% Triton-X 100 and 3 mM βME. Cells were lysed by sonication (Ultrasonic processor, Model-XL-2020, Germany) and lysate was centrifuged at 12,000 rpm for 30 min at 4°C. Supernatant was loaded on to Ni- nitrilotriacetic acid (NTA) column (Qiagen) pre-equilibrated with lysis buffer. Contaminating proteins were removed by subsequent three washes with washing buffers containing (50 mM Na_2_HPO_4_, 200 mM NaCl, 30 mM Imidazole), (50 mM Na_2_HPO_4_, 200 mM NaCl, 50 mM Imidazole) and (50 mM Na_2_HPO_4_, 200 mM NaCl, 100 mM Imidazole). Recombinant protein was finally eluted by elution buffer (50 mM Na_2_HPO_4_, 200 mM NaCl, 250 mM Imidazole). For all experiments the purified BmCRT was dialyzed overnight against 50 mM Na_2_HPO_4_, 150 mM NaCl, pH 7.5 buffer containing 10 mM EGTA and for removing EGTA it was dialyzed against 50 mM Na_2_HPO_4_, 150 mM NaCl, pH 7.5 buffer overnight. The protein concentration was determined by the method of Lowery et al [53]. Purity of eluted protein and its subunit mass was analyzed by 12% SDS-PAGE [54] and confirmed by Western blotting using anti-His antibodies.

### 2.4 Size exclusion chromatography (SEC)

Gel filtration was carried out using a Superdex 200 HR 10/300 column on an AKTA-FPLC. The column was calibrated with various standard molecular weight markers (Amershan). The gel filtration column was run in 50 mM sodium phosphate buffer (pH 7.6) containing 200 mM NaCl at a flow rate of 0.3 ml/min, with detection at 280 nm.

### 2.5 Production of polyclonal antisera to purified BmCRT

Male rabbit 3 to 5 weeks old (1–2 kg) was used for production of antibodies. The blood from the central ear artery was collected for preparation of preimmune sera. Three days later, rabbit was immunized subcutaneously with 250 µg of BmCRT in complete freund adjuvant (CFA). 21 days later the rabbit was injected with 250 µg of BmCRT in incomplete freund adjuvant (IFA) and a booster dose was given after one week. The rabbit sera was collected 10 days after the last (third) booster dose and the antibody titer was determined by ELISA in the serum.

### 2.6 Interaction of BmCRT with HuC1q protein

#### 2.6.1 Solid phase binding assay

Microtiter plate was coated with 100 µl/well of purified recombinant BmCRT (0 to 1.5 µg) diluted in carbonate buffer (15 mM of Na_2_CO_3_, 35 mM of NaHCO_3_, pH 9.6) for 6 h at room temperature. Control wells containing buffer and BSA. Following three washes with 0.05% Tween 20 in PBS (PBST), the wells were blocked with 150 µl of 5% skimmed milk in PBS for 2 h at 37°C. 0 to 5 µg of HuC1q (SIGMA) in 100 µl of 20 mM Tris-HCl (pH 7.4) containing 50 mM NaCl and 1 mM CaCl_2_ was added to each well and the plate was incubated at 4°C overnight. The plate was washed three times with PBST and 100 µl of rabbit anti-human C1q (at 1∶1500 dilution) was added to each well. After 2 h, wells were washed once again three times as described earlier and binding was detected by probing with HRP-conjugated goat anti-rabbit IgG (at 1∶3000 dilutions). The plate was kept at room temperature for 2 h followed by washing with PBST. Bound peroxides activity was measured by adding OPD (Orthophenyl diamine dihydrochloride, SIGMA) as substrate. The developed color was read at 490 nm in a microplate reader. The data are given as an average experiment ± standard deviation (SD).

#### 2.6.2 Haemolytic assay

The C1q-dependent haemolysis was measured by incubating 100 µl of C1q deficient serum [diluted 1∶40 in DGVB++ (isotonic Veronal buffered saline) containing 0.1 mM CaCl_2_, 0.5 mM MgCl_2_, 0.1% (W/V) gelatin and 1% glucose] with different concentrations of C1q for 30 min at 37°C. 100 µl of sheep erythrocytes (SRBC, 10^8^cells/ml) sensitized with rabbit anti-sheep RBC IgG (SIGMA) was added and further incubated at 37°C for 60 min. After centrifugation at 2500 rpm for 5 min, absorbance of 100 µl supernatant was measured at 405 nm for the determination of released hemoglobin. 1.5 µg of C1q which caused approximately 60–70% haemolysis, was incubated with 0–10 µg of BmCRT and BSA (as a control) in 100 µl of C1q deficient serum, for 30 min at 37°C. 100 µl of Ab-Sensitized erythrocytes (SRBC, 10^8^cells/ml) were added and further incubated at 37°C for 60 min. The unlysed cells were separated and absorbance of supernatant was measured at 405 nm for the determination of released hemoglobin. Normal human serum (NHS) was used as a positive control and BSA as a negative control. Lysis of erythrocyte was calculated considering the lysis of SRBC (10^8^) in the presence of water as 100%. Heamolytic activity of C1q in the presence of BmCRT is expressed as percentage of the total haemolysis [35], [42], [43].

#### 2.6.3 Interaction of BmCRT with HuC1q in presence of Ca^+2^

Microtiter plate was coated with 2 µg/well BmCRT in carbonate buffer and kept for 4 h at 37°C. Nonspecific binding sites were blocked by incubating with 5% skimmed milk for 1 to 2 h at 37°C and washing with PBST (PBS +0.05% Tween 20). HuC1q (0–3 µg/well) in TTBS buffer (0.05% Tween 20 and Tris-buffer saline containing 25 mM Tris +150 mM NaCl +2 mM KCl, pH 7.4) was added and incubated either in absence or presence of 5 mM CaCl_2_ or 20 mM EGTA overnight at 4°C. After washing with PBST plate was incubated for 2 h at 37°C with rabbit anti-human C1q antiserum (1∶1500 dilution). Interaction of both proteins was detected by probing with HRP-conjugated goat anti-rabbit IgG (1∶3000 dilution). OPD (Orthophenyl diamine dihydrochloride) was used as substrate for HRP. The color was measured at 490 nm in a microplate reader. The data are expressed as average experiments ± standard deviation (SD). Percentage of the effect of Ca^+2^ on the interaction of both proteins was calculated according to the method of Roumenina et al [55].

#### 2.6.4 Pull-down assay

130 µl Ni-NTA agarose beads in 1 ml binding buffer (10 mM Tris-HCl (pH 7.5), 150 mM NaCl, 10 mM imidazole, 0.1% Triton-X100 and 1 mM dithiothreitol (DTT), were incubated for 30–45 min at 4°C with 0.2 to 0.7 mg purified BmCRT as described previously [56]–[58]. Beads were washed three times with 2 ml of binding buffer containing 50 mM imidazole. Some hybridized beads were used for determination of binding of BmCRT by SDS-PAGE and remaining beads were incubated with human serum in 1 ml hybridization buffer (10 mM Tris-HCl (pH 7.5), 150 mM NaCl, 10 mM imidazole, 5 mM CaCl_2_, 0.1% Triton-X100, 1 mM DTT) for 1–2 h at 4°C. After three washing with hybridization buffer (30 mM imidazole), bound protein was eluted with elution buffer (containing 0.5 mM βME and 250 µl imidazole in binding buffer). Binding was visualized on 12% SDS-PAGE followed by Western blotting, using rabbit anti-human C1q (Abcam, at 1∶1500 dilution) and mice anti-BmCRT (at 1∶1000 dilution). HRP-conjugated anti-rabbit IgG (Abcam, at 1∶10,000 dilution) and anti-mouse IgG (Santa Cruz Biotechnology, at 1∶3000 dilution). Naked beads and C1q deficient human serum was taken as a negative control and immobilized BmCRT as a positive control.

#### 2.6.5 Competitive inhibition assay of BmCRT-C1q binding

Purified recombinant BmCRT (1 µg) was used for coating microtiter plate with 100 µl/well diluted in carbonate buffer (15 mM of Na_2_CO_3_, 35 mM of NaHCO_3_, pH 9.6) for 4 h at room temperature. Following three washes with 0.05% Tween 20 in PBS (PBST), the wells were blocked with 150 µl of 5% skimmed milk in PBS for 2 h at 37°C. 1 µg of C1q (SIGMA) was preincubated with human IgG (0.25 to 5 times excess, w∶w), human SAP (10 to 400 times excess, w∶w) and BSA (control) diluted in 100 µl of 20 mM Tris-HCl (pH 7.4), 50 mM NaCl and 1 mM CaCl_2_ for 2 h at room temperature before addition to each well and was allowed to incubate for overnight at 4°C. The plate was washed three times with PBST and 100 µl of rabbit anti-human C1q (at 1∶1500 dilution) was added to each well. After 2 h, wells were washed once again three times as described earlier and binding was detected by probing with HRP-conjugated goat anti-rabbit IgG (at 1∶3000 dilutions). The plate was kept at room temperature for 2 h followed by washing with PBST. Bound peroxides activity was measured by adding OPD (Orthophenyl diamine dihydrochloride, SIGMA) as substrate. The developed color was read at 490 nm in a microplate reader. The data are expressed as an average experiment ± standard deviation (SD).

### 2.7 Expression of BmCRT in filarial parasite

Expression of BmCRT in parasite at different stages of lifecycle was determined with the help of Western blotting, using polyclonal antibody generated in rabbit. 45 µg of adult, L3, Mf protein and 180 µg E/S products of adult worm were resolved on 12% SDS-PAGE and protein bands were transferred onto the nitrocellulose membrane at 4°C for 3–4 h and blocked by 5% skimmed milk in PBS for overnight at 4°C. After three washings with PBST buffer membrane was incubated with rabbit anti-BmCRT (diluted, 1∶1000) for 2 h, then followed by incubation with HRP conjugated goat anti-rabbit IgG (SIGMA, at 1∶3000 dilutions) for 2 h. Finally, bands were visualized by the addition of DAB (3, 3 Diaminobenzidine tetrahydrochloride) as substrate.

### 2.8 Circular Dichroism analysis

CD spectropolarimetry was performed with 0.2 mg/ml BmCRT in 50 mM NaH_2_PO_4_ and 100 mM NaCl. The secondary structure of BmCRT was monitored in the far-UV region between 260 and 190 nm at 25°C with JascoJ810 spectropolarimeter using a cell with a 0.1 cm path length. A scanning with buffer without protein was recorded under identical conditions to determine the background spectra. The far-UV CD spectrum was analyzed for secondary structure content by use of K2D software [59].

### 2.9 Modeling

#### 2.9.1 Template Searching and Sequence alignment

The amino acid sequence of *B. malayi* calreticulin (Bm1_23560) was retrieved from the sequence database of NCBI (www.ncbi.nlm.nih.gov) (ID: XP_001896170.1). The three-dimensional structures of calreticulin precursor were not available in Protein Data Bank (PDB); hence the present exercise of developing the 3D models of the calreticulin precursor of *B. malayi* was undertaken. To find suitable templates for homology modeling BLASTP search was performed against the Brookhaven Protein Data Bank with the default parameters. Based on the maximum identity with high score and lower e-value, the structure of Arm Domains of calreticulin (PDB code: 3RG0: Chain A) was selected as template for building three dimensional structure. The sequence alignment between Bm1_23560 and 3RG0 was carried out using the CLUSTAL W (http://www.ebi.ac.uk/clustalw) program.

#### 2.9.2 3D Structure generation

The commercial version of Prime V3.0 [60] was used for 3D structure generation based on the information obtained from sequence alignment. Alignment of sequence and template was morphed and the secondary structure was predicted. Finally using the prime algorithm the refined structure was generated and minimized until the average root-mean-square-deviation (RMSD) of the non-hydrogen atoms reached 0.3 Å using the protein preparation wizard [61]. The modeled structure was then superimposed on the crystal template without altering the coordinate systems of atomic position in the template. The residue profiles of the three-dimensional models were further checked using VERIFY3D [62]. In order to assess the overall stereo chemical quality of the modelled protein, Ramachandran plot analysis was performed using the program PROCHECK. Quality of generated models was evaluated with by QMEAN, ANNOLEA, GROMOS and PROSA analysis. The structures of template and target sequence were aligned using the Chimera – Molecular Visualization program. The modeled structure BmCRT and HuC1q binding site information has not yet been reported; hence we hope that the prediction of these binding site regions will enhance the structural information of BmCRT-C1q complex. Here the possible binding sites were predicted based on interaction specificity region through sitemap with OPLS-2005 force field.

#### 2.9.3 Protein-Protein Docking

A geometry-based molecular docking algorithm called Patch Dock (http://bioinfo3d.cs.tau.ac.il/PatchDock) was used for docking the predicted three dimensional models of calreticulin and crystal structure of complement system protein C1q [63]. The Patch Dock server predicts the docked transformations that produce good molecular shape complementarity. The algorithm divides the Connolly dot surface representation of the molecules into concave, convex, and flat patches. The patches were matched according to their complementarities in order to generate different transformations. A default value of 4 Å was used for clustering and redundant solutions were discarded by RMSD clustering. The Patch Dock output generates the geometric score, desolvation energy, interface area size, and the actual rigid transformation of the solutions. Twenty solutions, out of 60 predicted complexes, were sorted according to their geometric shape were again refined through the fire dock server [64]. The complementarities scores were analyzed for identifying the residues involved in the protein-protein interface.

#### 2.9.4 Potential Biological complex and Binding energy calculation

The structure of protein and protein assemblies were experimentally accessible through the interpretation of images obtained by the diffraction of X-rays by a protein crystal. The proteins structures solved through X-Ray or NMR are lacking in the information of interactions that are specific in binding to other substances like macromolecules, small molecules and metals. These interactions are playing the vital role in formation of biological complex and stabilizing the complexes. To check the docked protein-protein complex as potential biological complex, we used the DiMoVo server [65]. Potential biological complex was predicted using the SVM methods and values above 0.5 indicated that the complex obtained was biologically potent. We also checked the role of metal ion in the participation of biological complex through presence and absence of metal ion in the complex and their energy difference was noted using the binding energy calculation (MM/GBSA approach). Binding energy was calculated by the following equations.



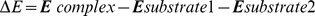



(E_complex_, E_substrate1_, and E_substrate2_) are the minimized energies of the protein-protein complex, protein and another respectively [66].

#### 2.9.5 Molecular Dynamics Simulation

Molecular dynamics (MD) simulations were performed using the GROMACS 4.5 package [67]. The GROMACS program package (http://www.gromacs.org) adopting the OPLSAA force field parameters were used for EM and MD simulations. The BmCRT (Homology model), C1q (crystal structure) and the protein-protein complex (BmCRT–C1q) were analyzed in three separate system to obtain the stable conformation of the protein and protein-protein complex for analyzing three dynamic behavior of these structures. The bad contacts from the 3D structure of the proteins were refined and solvated with the solvent [68]. The system was further relaxed by energy minimization and for the MD simulation studies, the structures were solvated using the TIP3P water model and the solvated structures were energy minimized using the steepest descent method, terminating when maximum force was found smaller than 100 KJ/mol-1/nm-1 [69]. All the simulations were performed in the NPT ensemble at constant temperature (300 K) and pressure (1 bar) with a time step of 2 fs. NVT were performed for 1ns (nanoseconds) and the minimized structure were equilibrated with timescale of 10 ns (nanoseconds). Trajectory conversion and RMSD scripts were used for analysis of MD simulation [70].

#### 2.9.6 Visualization of Protein – Protein interactions

The residual interactions between the predicted three dimensional model of calreticulin and C1q were visualized through the academic version of chimera. Here the color intensity for interactions was visualized clearly and exported for results [71]. Bonded and Non-bonded interacting residues between the protein-protein interactions were examined through PDBSUM and chain wise interactions are visualized.

### 2.10 Statistical Analysis

Each experiment was performed in triplicate and the results are expressed as mean ± SD. Data were analyzed using two way analysis of variance (ANOVA) with the help of statistical software PRISM 5 (Graphpad software). The criterion for statistical significance between the groups was as follows: p value, 0.05 was considered significant and marked as *, 0.01 as highly significant and marked as **, 0.001 as very highly significant and marked as ***.

## Results

### 3.1 Cloning and sequence alignment

**Figure 1:**
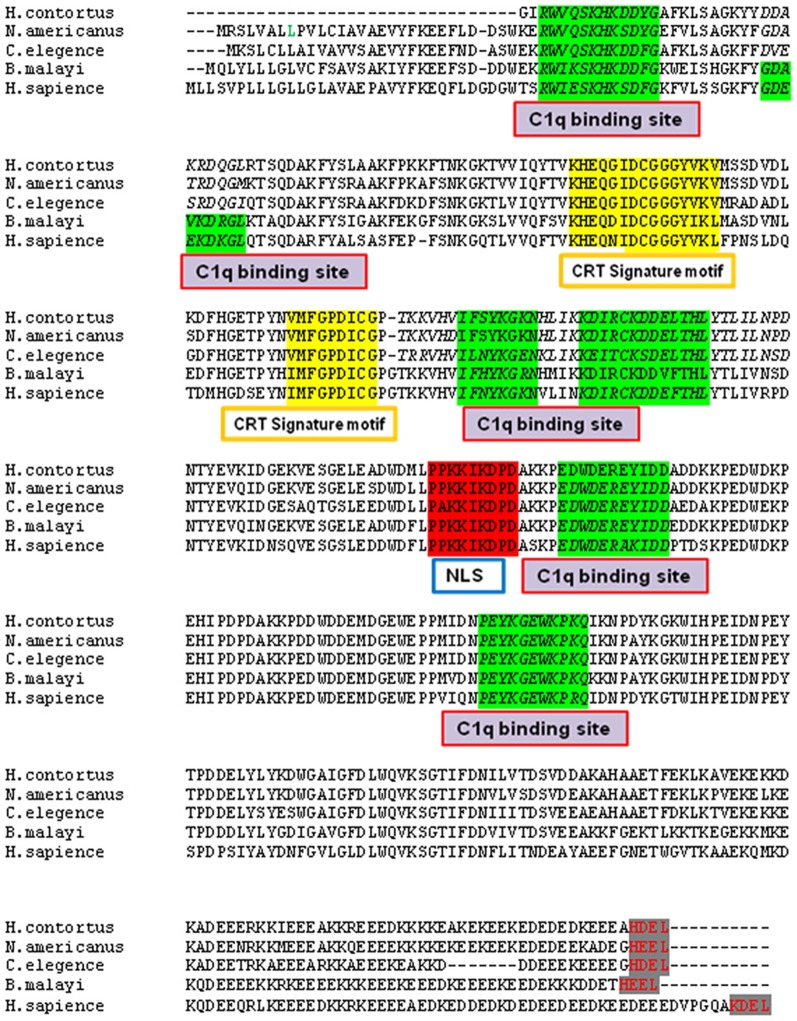
Multiple Amino acid Sequence alignment of BmCRT with parasites and human CRT. Six C1q binding sites (green shading), C terminal ER targeting sequences (Grey shading), Putative nuclear localization signal site (Red shading) and CRT signature motifs (yellow shading) are indicated.

BmCRT cDNA contains an open reading frame of 1250 bp, which encodes a protein with a predicted molecular mass of 46 kDa. Figure 1 shows the amino acid sequence alignment (http://www.genome.jp/tools-bin/clustalw) of BmCRT with CRT of other nematodes and human. The BmCRT contains conserved regions viz two signature motifs of CRT super family, a Endoplasmic reticulum (ER) retention signal with an acceptable variant, which functions in the retrieval of ER resident proteins [26], a putative nuclear localization signal site and highly conserved C1q binding motifs. BmCRT contains six C1q binding sites like Human CRT, while only five C1q binding sites have been reported for others [42], [43].

Our BLAST sequence analysis of BmCRT showed 56, 62, 60, 57 and 38% homology with human, *H. contortus, N. americanus, C. elegans* and *T. cruzi* respectively and more than 70% with other filarial parasits (*W. bancofti, Loa-loa, O. volvulos*).

### 3.2 Over-expression and purification of *Bm*CRT

**Figure 2:**
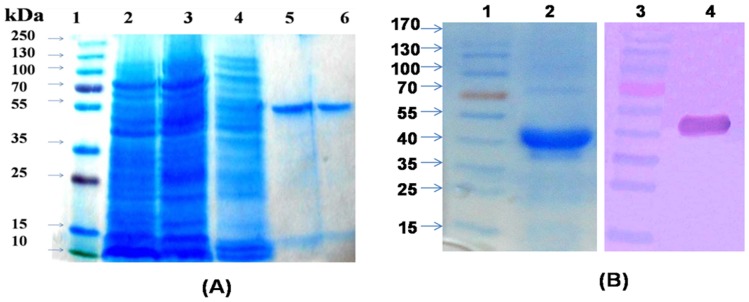
Purification of recombinant BmCRT protein. (A) 12% SDS-PAGE analysis of purified recombinant BmCRT. Lane 1: molecular weight markers, Lane 2: soluble fraction of induced cells, Lane 3: flowthrough fraction, Lane 4: 30 mM imidazole wash, Lane 5–6: protein eluted with 250 mM imidazole. Single band showing 46 kDa purified rBmCRT protein. **(B) Western blot analysis of purified rBmCRT using anti-His antibody.** Lane 1–3: molecular weight marker, Lane 2: Coomassie staining of 25 µg of purified rBmCRT on 12%SDS-PAGE, Lane 4: purified rBmCRT probed by anti-His antibody.

**Figure 3:**
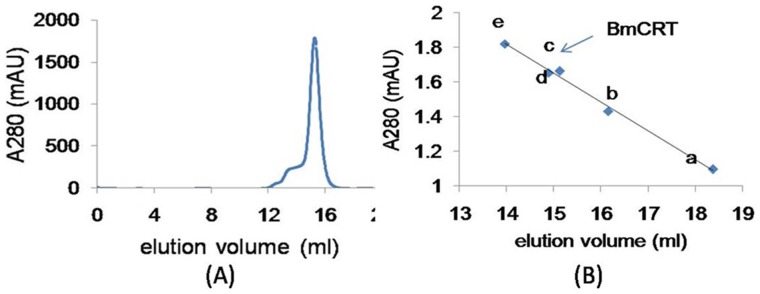
Determination of native molecular mass and oligomeric form of BmCRT. (**A**) Protein fraction on Superdex-G200 10/30 column by AKTA was carried out. Protein was eluted with 50 mM Na_2_HPO_4_+150 mM NaCl buffer (pH 7.5), at 15.13 ml elution volume. The flow rate was 0.35 ml/min. (**B**) The column was calibrated with standered molecular weight markers: (a) Ribonulease (12.5 kDa) (b) Carbonic anhydrate (27 kDa) (d) Ovalbumin (45 kDa) (e) Albumin (66 kDa) and arrow indicate (c) BmCRT (46 kDa).

*Brugia malay*i cDNA was used as a template for PCR amplification of BmCRT gene. 1.2 Kb PCR amplified product was cloned in pET28a^+^ expression vector and expressed in BL21 (DE3) pLysS *E. coli* strain. The induction was carried out with 1 mM IPTG at 20°C for 18 h. This resulted in the expression of 46 kDa protein and the expressed protein was purified by Ni-NTA affinity chromatography (Figure 2A). A subunit molecular mass of 46 kDa was obtained for expressed and purified BmCRT by SDS-PAGE, at the same time, expression of His-BmCRT was confirmed by Western blotting using anti-His antibody (Figure 2B). The size exclusion chromatography showed a single peak eluting at 15.6 ml (Figure 3A). The sephadex-200 column was calibrated with standard molecular markers and BmCRT was found to have monomeric molecular mass of 46 kDa (Figure 3B).

### 3.3 BmCRT interaction with HuC1q

**Figure 4:**
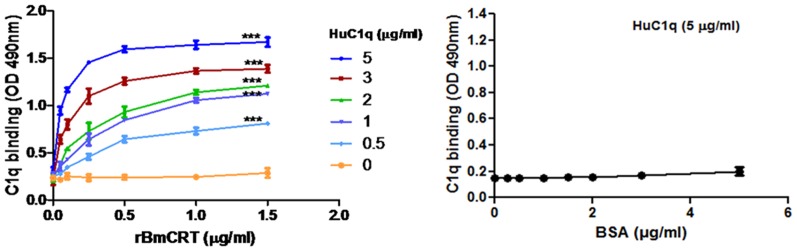
Dose dependent interaction between BmCRT and HuC1q by solide-phase binding assay. Microtiter plate was coated with 0–30 µg of rBmCRT (**A**) or BSA (**B**) in carbonate buffer. After blocking with 5% skimmed milk the plate was incubated with human C1q and rabbit anti-human IgG Abs were used to detect the interaction of both protein as mentioned in method section. No binding of C1q was observed with BSA (control). The significant between BSA (or 0.0 concentration of C1q) and different concentration of C1q were analyzed by two-way ANOVA (***P<0.001). Assay was performed in triplicates. Bar represent the standard deviations of the mean.

C1q plays an important role in immune system by binding with IgG, IgM and CRP [10]. Interference of this binding by CRT protein was responsible for inhibition of C1q function by the formation of CRT-C1q complex, which leads to autoimmune diseases in human and establishment of parasite infection in host [16], [20], [36]. To assess the interaction between BmCRT and C1q, direct binding experiments were conducted in microtiter plate under the physiological condition. Rabbit anti-humanC1q antibody was used to confirm that C1q is bound to BmCRT coated plates in a dose dependent manner while no binding was observed in BSA coated plate (Figure 4). Since binding ability of BmCRT to C1q may interfere with C1q-mediated functions, this interference of BmCRT was assayed by C1q-mediated haemolysis.

### 3.4 Inhibition of C1q haemolytic activity

**Figure 5:**
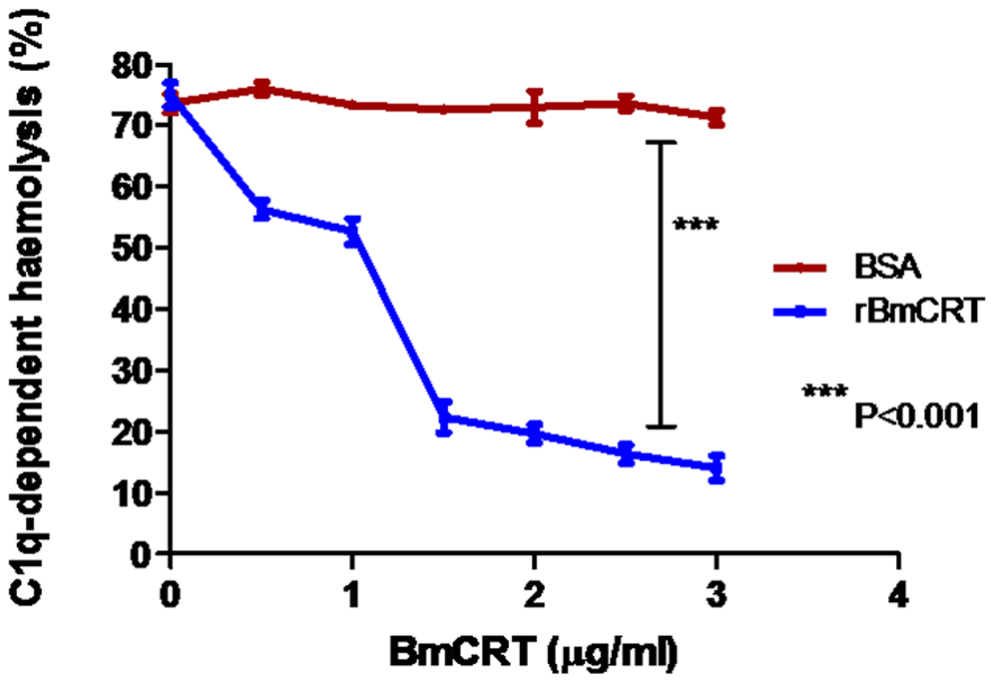
BmCRT prevent C1q-dependent haemolysis of Ig-sensitized erythrocytes. Recombinant BmCRT inhibits C1q-dependent haemolysis while BSA (control) shows no effect on this when incubated with C1q deficient serum together with C1q as described in material and method section. Percentage of cell lysis was calculated with reference to 100% lysis of the cells in water. The significant were analyzed by two-way ANOVA (***P<0.001). Assay was performed in triplicates. Bar represent the standard deviations of the mean.

Classical complement system is activated by the binding of C1q to the F_c_ regions of immune-complex (IC) containing IgM or IgG antibody (IgG-IC). Binding of BmCRT to C1q affected the classical-complement dependent haemolysis activity. Deactivation of this system by BmCRT was determined through C1q dependent haemolysis. The results showed that 1.5 µg C1q caused 60–75% haemolysis, but addition of varying concentration of BmCRT (0–5 µg) decreased haemolysis in a dose dependent manner from 75% to 20% (Figure 5). BSA showed no effect while NHS showed 78% haemolysis without addition of BmCRT or C1q. C1q deficient human serum also caused no haemolysis as reported by Kishore et al [35]. These results indicated that the interaction of both proteins (BmCRT-C1q) is responsible for blockage of classical complement system.

### 3.5 Effect of Ca^+2^ on BmCRT-C1q interaction

**Figure 6:**
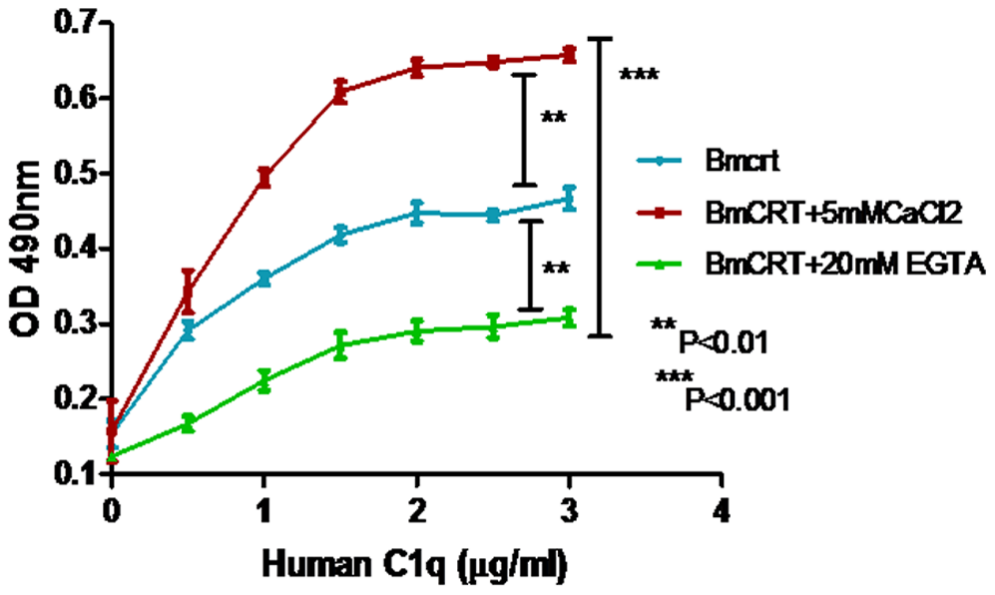
Presence of Ca^+2^ is essential for the interaction of BmCRT - HuC1q. Microtiter plate was coated with BmCRT and after blocking the remaining protein-binding site with skimmed milk wells were incubated overnight with a HuC1q as mentioned in method section. Effect of Ca^+2^ on the binding of both proteins was measured at 490 nm. The data are presented as means ± SD; n = 3. ** P<0.01, ***P<0.001.

Since Ca^+2^ is essential for C1q stability and its function [10], [55] while BmCRT is a Ca^+2^ binding protein, the possibility exists that Ca^+2^ binding tendency of BmCRT might be involved in inhibition of C1q function. To address this question, calcium free BmCRT and C1q saturated with calcium were used to study BmCRT-C1q interaction. The studies were carried out in TTBS buffer only, in 5 mM CaCl_2_ and in 20 mM EGTA as shown in figure 6. This result showed that the interaction of BmCRT with C1q was increased upto 40% in presence of calcium. Since Ca^+2^ binding is the intrinsic property of the C1q and addition of extra Ca^+2^ is found to have no effect on its binding ability as reported by Roumenina et al, 2005 [55], the increased binding of C1q with BmCRT may be due to some conformational changes in BmCRT after binding with Ca^+2^ (data not shown) exposing its C1q binding sites. But with apo C1q (upon addition of 20 mM EGTA) only 10–20% binding was observed (Figure 6), this may be due to the fact that Ca^+2^ stabilize the heterotrimetic structure of C1q, required for its target recognition [10]. Apo C1q showed similar binding with both holo and apo forms of BmCRT (data not shown).

### 3.6 Pull-down assay

**Figure 7:**
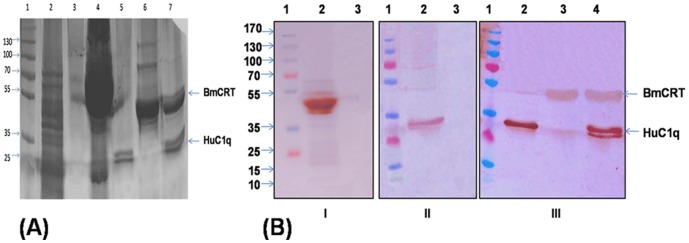
Pull-down assay demonstrating *in vitro* protein-protein interaction between BmCRT and HuC1q. (A) Visualization of BmCRT-HuC1Q complex formation on 12% SDS-PAGE. Lane 1: Marker, Lane 2: Flow through after binding rBmCRT, Lane 3: washing with binding buffer, Lane 4: NHS after passing through rBmCRT bound beeds, Lane 5: molecular weight marker of human C1q (Sigma), Lane 6: molecular weight marker of rBmCRT, Lane 7: eluted product after passing NHS through rBmCRT bound beeds, two bands at 46 kDa (BmCRT) and 25 kDa (C1q) indicates BmCRT-C1q interaction. **(B) Western blot analysis of the interaction between BmCRT-C1q by pull-down assay using mice anti-BmCRT and rabbit anti-human C1q antibodies.** (I) Western blot to recognize immobilized BmCRT (Bait), using mice anti-BmCRT antibodies: Lane 1: Marker, Lane 2: covalently loaded beads with bait, Lane 3: Naked beads. (II) Western blot to recognize HuC1q (prey), using rabbit anti-humanC1q antibodies: Lane 1: Marker, Lane 2: NHS pass through immobilized bait, Lane 3: NHS pass through naked beads. (III) Western blot to visualized the interaction of both proteins, using mice anti-BmCRT and rabbit anti-humanC1q antibodies: Lane 1: Marker, Lane 2: NHS only, Lane 3: C1q deficient normal serum pass through immobilized beads, Lane 4: NHS pass through immobilized BmCRT.

The *in vitro* pull-down assay was used for visualization of the interaction of purified BmCRT with human complement protein C1q on 12% SDS-PAGE (Figure 7A) and confirmed by Western blotting using specific antibodies for both proteins. Purified BmCRT was dialyzed (against binding buffer) and used as bait for normal human serum (NHS). When NHS was passed through BmCRT bound beads, C1q was detected along with BmCRT but when C1q deficient human serum was passed then no C1q was detected along with BmCRT (Figure 7B III). C1q was also not detected after passing NHS through naked beads (Figure 7B II). This confirmed the binding of both proteins (Figure 7 B).

### 3.7 Identification of BmCRT binding region on HuC1q

**Figure 8:**
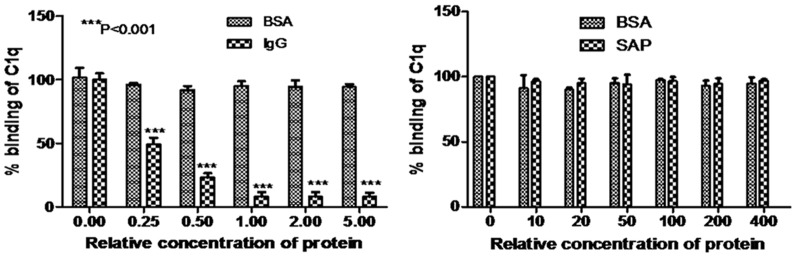
BmCRT binds at the globular head region of C1q. (A) IgG inhibits binding of C1q to immobilized BmCRT. Microtiter plate was coated with BmCRT(1 µg/ml) in corbonate buffer (pH 9.6). C1q (1 µg/ml) was preincubated with 0.25 to 5 times excess (w∶w) IgG for 2 h at room temperature before addition to wells and and incubate overnight at 4°C. Binding of BmCRT with C1q in presence of IgG was observed by taking OD at 490 nm as mentioned in method section. **(B) SAP shows no effect on BmCRT-C1q binding.** Preincubated C1q (1 µg/ml) with 20 to 400 times execces (w∶w) SAP for 2 h was added in BmCRT (1 µg/ml) coated wells and binding of BmCRT with C1q was observed at 490 nm. BSA taking in both assays as a control, show no effect on BmCRT-C1q complex formation. The results were analyzed by two-way ANOVA (***P<0.001). Assay was performed in triplicates. Bar represent the standard deviations of the mean.

In order to localize the BmCRT binding site with C1q, BmCRT-C1q interaction was performed in the presence of IgG and SAP. It is reported that IgG binds at the head region of C1q while SAP binds at its collagen-like tail [45], [72]. Interestingly our results show that the binding of C1q to immobilized BmCRT was strongly inhibited by IgG in dose dependent manner, while no effect was observed on this binding by SAP at its maximum concentrations (Figure 8). This suggests that IgG and BmCRT bind at the same site on C1q, thus supporting the proposed interaction of BmCRT with head region of C1q not with its collagen region. No binding was observed between IgG and SAP with BmCRT (data not shown).

### 3.8 Expression of BmCRT in different stages of parasite lifecycle

**Figure 9:**
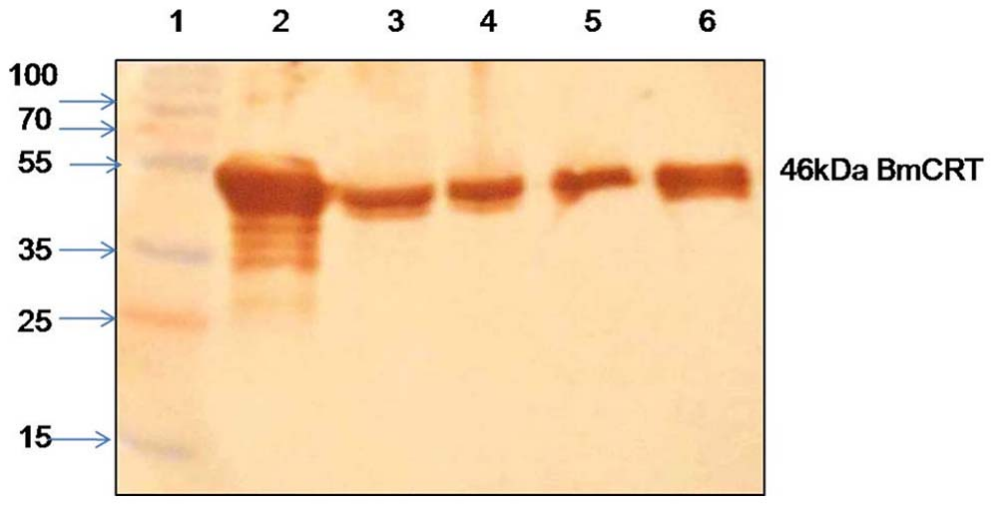
Western blot to confirm the expression of BmCRT at different stages of *B. malayi* life cycle using sera of rabbit immobilized with recombinant BmCRT. 46 kDa band of BmCRT was detected in all stages as well as in E/S product of adult worm Lane 1: Marker, Lane 2: purified recombinant BmCRT, Lane 3: infectve stage (L3) lysates, Lane 4: pathogenic stage (Adult worm) lysates, Lane 5: E/S products of Adult worm, Lane 6: Discharge stage (Mf) lysates.

The antibodies against the recombinant BmCRT were used for analysis of BmCRT expression in different life stages of parasite and its presence in E/S products. The antibody titer against BmCRT in the rabbit serum was found to be 1∶2,50,000 and the antibodies specifically recognized the recombinant BmCRT as well as CRT in adult, L3, Mf lysates and E/S products of adult worms (Figure 9). Its presence in E/S products indicated that BmCRT is a secretory protein, which is expressed in different life stages of the human filarial parasite.

### 3.9 Secondary structure of BmCRT

**Figure 10:**
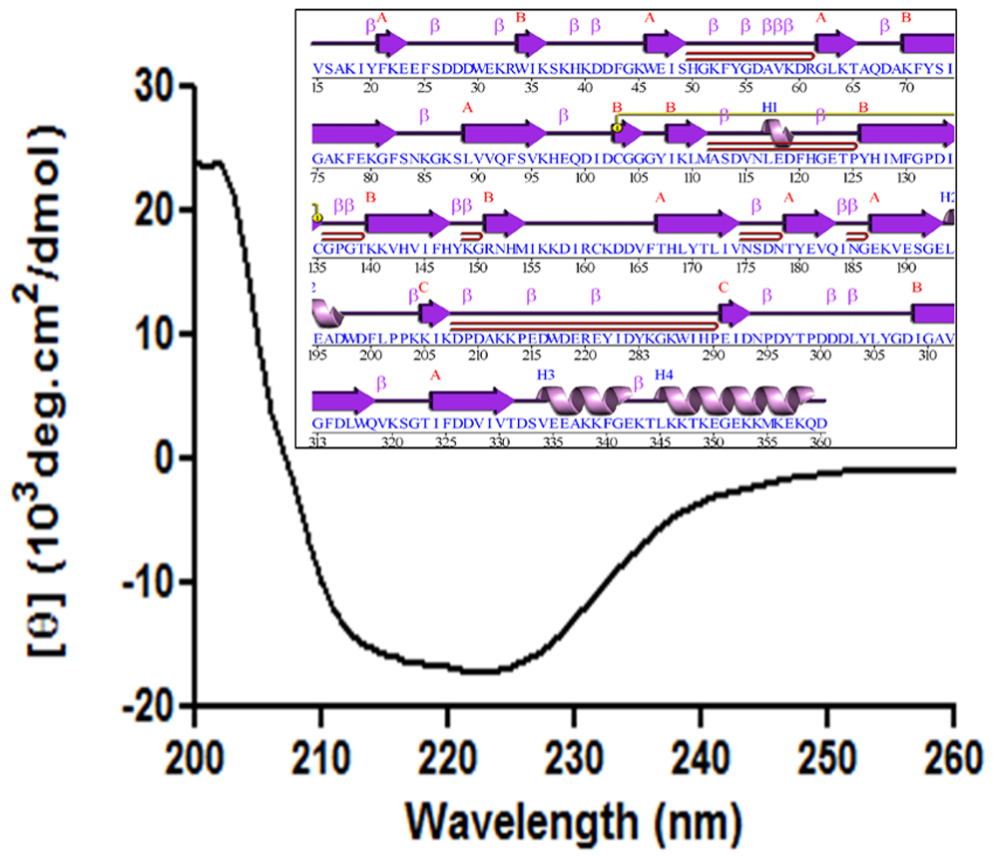
Far-UV CD spectra of BmCRT. CD measurement was caried out in JascoJ810 spectropolarimeter, protein spectra were recorded between 190–250nmwavelength at 25°C. The spectrum was analyzed by use of K2D software. Inset showed Schematic diagram of BmCRT modeled protein, the ‘wiring diagram’ shows the protein's secondary structure elements (α-helices and β-sheets) together with various structural motifs such as β- and γ-turns, and β-hairpins with their corresponding amino acid residues.

The far-UV (260–190 nm) CD spectrum was utilized for obtaining the information about the secondary structure of the protein. The BmCRT spectrum showed negative peak at 222 nm (Figure 10). BmCRT retains all secondary features with 49.6% α-helix, 9.6% β-sheet and 43.6% random coil. The ellipticity at 222 nm was approximately −17.0×10^3^ deg cm^2^ dmol^−1^.

### 3.10 Modeling

#### 3.10.1 Homology modeling and binding site prediction of BmCRT

**Figure 11:**
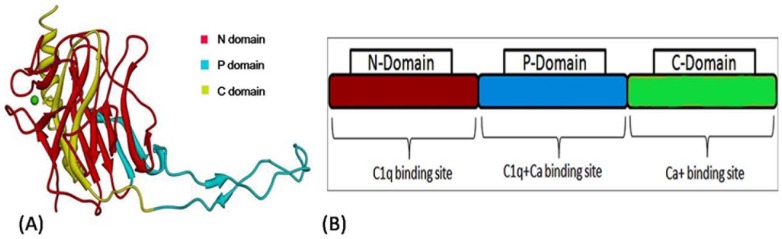
Structure and Domain pattern of BmCRT. (**A**) 3D structure of BmCRT showing N-Domain (Red), P-Domain (Blue) and C-Domain (Yellow). (**B**) BmCRT domains-interaction allocation.

**Figure 12:**
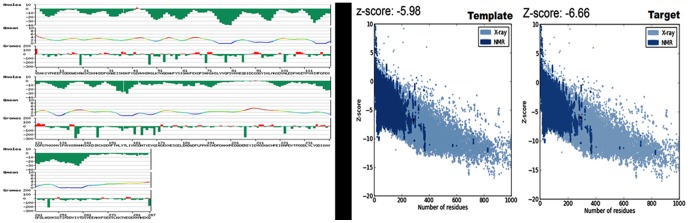
Validation of Protein Models. (**A**) Graphical representation of ANOLEA, QMEAN and GROMOS analysis of modeled BmCRT protein structure. (**B**) ProSA analysis indicated that the overall interaction energy of the model was measure in Z-Score -6.86 kcal/mol and template was −6.41 kcal/mol.

**Figure 13:**
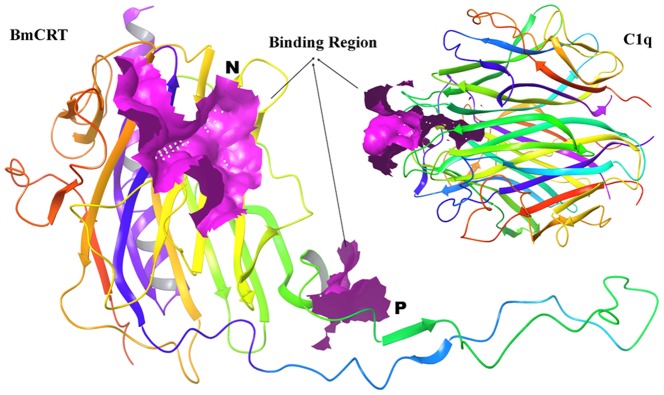
Predicted binding site region of BmCRT showing pink colored surface located in N and P domains, and structure of Human C1q, showing pink colored surface in the head region located in top portion of all three chains.

The 3D and crystal structure of BmCRT are not available hence for homology modeling macromolecular structure of calreticulin Arm domains was utilized (PDB ID 3RG0). The BmCRT Sequence highly matched in terms of both phylogeny and functional similarity, showing 59% similarity with calreticulin Arm domains (Figure S1). Prime search for family of the source protein using the phylogenetic analysis predicted to be the calreticulin family proteins. As the source and template sequence are having the same function of calreticulin and so choosing the 3RG0 as template structure will provide more accurate 3D structure of BmCRT. Most of the functional residues are conserved in both template and target sequences which provide additional support for homology model protein will also have similar functions (Figure S1). On comparing the template with functionally similar structures deposited in the PDB ID 3RG0 and 3POS calreticulin's, we noticed few amino acids changes has not modulated the calreticulin function. The main reason for not using the another similar protein structure namely “X-ray structure of the human calreticulin globular domain reveals a peptide-binding area and suggests a multi-molecular mechanism” reported in the PDB ID- 3POS is due to the lack of similarity in tail regions. The sequence of ID: XP_001896170.1 is searched for template and we found that 3RG0 provides maximum covering of sequence in place of 3POS. The structural comparison of globular domain of the human CRT and homology modeled BmCRT is provided as Supplementary information (Figure S2). Here, the brown color represents the structure of 3POS and blue color represents the modeled structure of BmCRT. This clearly visualizes the lack of tail region in the 3POS and choosing of 3RG0 is reasonable for modeling the complete structure of BmCRT. 3D model is generated using the predicted secondary structure, which is shown in (Figure 11). The secondary structure of protein resembled conformation of template structure and was dominated by alpha helix, beta sheet and loop region and matched with the results obtained from CD spectrum. The modeled structure of BmCRT morphed with 3RG0 protein shows a deviation with a RMSD value of 0.31 Å (Figure S3). The electrostatic potential shows that the charge regions were very similar with template protein and the tail like structure present in BmCRT showed more negative regions which might actively participate in binding interactions (Figure S4). Analyzing the generated 3D structure for structure verification by Ramachandran plot values of 84.5 of core, 14.7% of allowed regions, 0.4% in general and 0.4% in disallowed regions were obtained (Figure S5). The overall quality of the modeled protein was evaluated using the errat value and it showed the value of 85.824 (Figure S6). The analysis of QMEAN, ANOLEA and GROMOS structure assessment tools reveal that the global and local properties of BmCRT protein model generated by automated SWISS-MODEL server using macromolecular structure of calreticulin Arm domains (3RG0) as template is reliable and shows the values of structure is allotted in acceptable range (Figure 12a). Additionally, PROSA server is used to check potential errors in predicted 3D models of protein. The Z-score indicated the overall quality of model and also measure the deviation of total energy in predicted model with respect to energy distribution from random conformations. The Z-score of the template is −5.98 kcal/mol and of target is −6.66 kcal/mol and it indicates that the modeled structure is much similar to template structure (Figure 12b). Overall the quality of 3D models was quantified by Procheck, Swiss Model server and PROSA reflects the quality of the 3D model is reliable and it can be used for further analysis. Theoretically, the binding regions of the BmCRT are investigated by using the Sitemap V3.0. Sitemap's proven algorithm for binding site identification and evaluation can help researchers to locate binding sites with a high degree of confidence and predict the binding ability of those sites. In visualizing the sitemap predicted binding site region, the binding site is located in the N and P domain (particular region focused as pink colored surface) for the model structure of BmCRT, as shown in the figure 13. For HuC1q, the binding site region is present in the top portion and exactly in between all three chains (A, B, C). Majority of binding site region is placed near the B chains as shown in the figure 13.

#### 3.10.2 Protein-Protein Interactions

**Figure 14:**
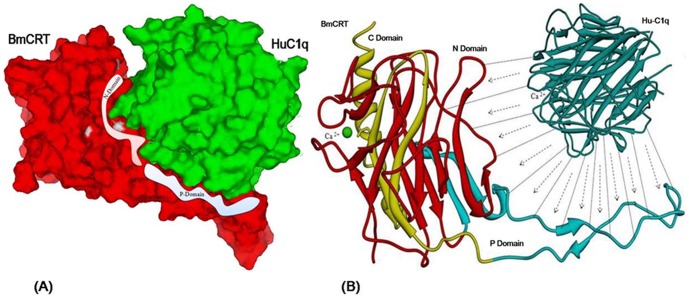
Protein-protein interaction between modeled BmCRT and Human C1q (1PK6). (**A**) Protein-Protein complex showing surface interactions between BmCRT and C1q protein.(**B**) BmCRT Domain seeking interactions with Human C1q.

**Figure 15:**
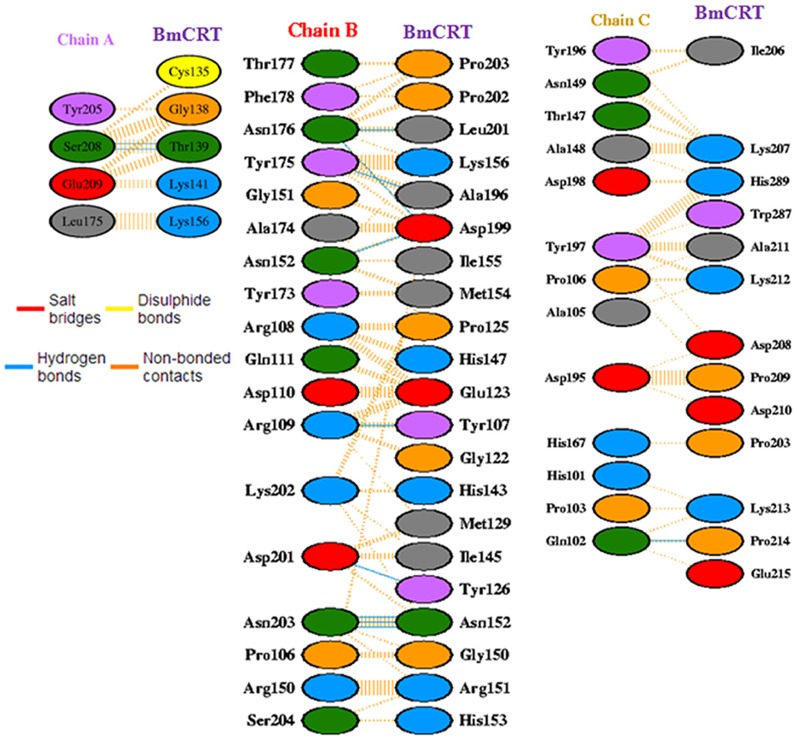
Protein-protein interactions of BmCRT with Human C1q (Chain A, B, C). All three chains (A, B, C) of HuC1q was involved in complex (BmCRT-C1q) formation with N and P domain of BmCRT.

Many biological function of protein depend upon the formation of protein-protein complexes. So here we studied the protein-protein interaction of BmCRT with C1q. The HuC1q structure is composed of three chain structures (A, B and C). The region of metal binding cleft is recognized to be the site for protein interaction and results obtained with BmCRT showed that the interactions take place near the metal ion binding region. When viewing the interactions, the C1q showed the involvement of N and P-domain residues of BmCRT in formation of the Protein-protein complex (Figure 14). Especially the N-domain amino acids Cys^135^, Gly^138^, Thr^139^, Lys^141^, and Lys^156^ are involved in the interactions with A chain of C1q. Some of the N-domain amino acids Ile^155^, Lys^156^, His^147^, Glu^123^, Tyr^107^, Gly^122^, His^143^, Ile^145^, Pro^125^, Tyr^126^, Met^129^, Asn^152^, Gly^150^, Arg^151^, His^153^ and Met^154^ are interacted with B chain of C1q. None of the N-domain amino acids made any contact with the C chain of C1q. Along with N-domain, some of the P-domain amino acids Pro^203^, Pro^202^, Leu^201^, Ala^196^ and Asp^199^ makes contact with B-chain and amino acids Ile^206^, Lys^207^, His^289^, Trp^287^, Ala^211^, Lys^212^, Asp^208^, Pro^209^, Asp^210^, Lys^213^, Pro^214^, Glu^215^ with C-chain and also played a role in interactions with both hydrogen bonding contacts and non-bonding interactions (Figure 15). The bonding interaction details between BmCRT and C1q are provided in table 1.

**Table 1:** BmCRT and Human C1q interaction details showing number of hydrogen bonds and non bonded contacts.

Interactions	No. of interface residues	Interface area (Å^2^)	No. of salt bridges	No. of disulphide bonds	No. of hydrogen bonds	No. of non-bonded contacts
C1q (A)—BmCRT	4∶5	223∶206	-	-	2	25
C1q (B)—BmCRT	18∶21	1032∶983	-	-	9	194
C1q (C)—BmCRT	13∶13	606∶579	-	-	1	84

Calcium ion present in the C1q protein functions as neutralizing components and the surrounding regions of ions are negative charged. These metal ions participate in the rigidity or compactness of the C1q protein (Figure S7). Protein-Protein interaction results also confirmed that BmCRT binds at the calcium bouning regions. On visualizing the metal interactions, initially the C1q protein residue namely Asp^172^, Tyr^173^, Gln^177^ and Gln^179^ are having the crystallographic contacts (bonding interactions) with Ca^+2^ ions (Figure S8A). After BmCRT binding, the bonding networks of metal shows that the Gln^177^ was not able to interact with the Ca^+2^ ion. This may be due to the collision of two big macromolecular structures which makes the amino acid Gln^177^ to lose its original contact due to molecular level changes occurs in HuC1q (Figure S8B). In order to check the importance of Ca^+2^ ions in the protein function, we checked the biological potency and binding energy scoring values in presence and absence of metal ion. The values above 0.5 were considered as the potentially better for biological complexes, which are predicted to have more correlation with natural biological protein-protein complexes. When viewing the scoring values in the presence of metal in the complex it showed the value of 0.8 and in absence of metal ion 0.5 values was observed. The DiMoVo server results strongly predicted that the metal ions are crucial for the protein-protein complex formation between the BmCRT and C1q. Along with this; we computed the binding energy calculation of the complex in the presence and absence of metal ion. The binding energy between protein-protein was also investigated both in the presence and absence of the metal ion. In the absence of metal ion the energy values are suddenly decreased showing its non- suitability for the complex formation. The energy between the complex and two proteins varied in the presence and absence of metal ion and also resulted in rupturing of interaction bridges between the two proteins. Both the DiMoVo server and binding energy calculation studies reports that, removal of metal ion from the C1q will deduce the scoring values in this protein-protein complex (Refer table 2).

**Table 2:** Scoring Values of potential biological complex in presence and absence of metal ion.

	With Metal	Without Metal	
Potential Biological complex rating	0.85405	0.58214	This score was computed using DiMoVo 0.5 with 27 descriptors.
Binding energy	−42.5518	−32.8510	Computed by MM/GBSA approach

#### 3.10.3 Molecular Dynamics Simulation (MDS)

**Figure 16:**
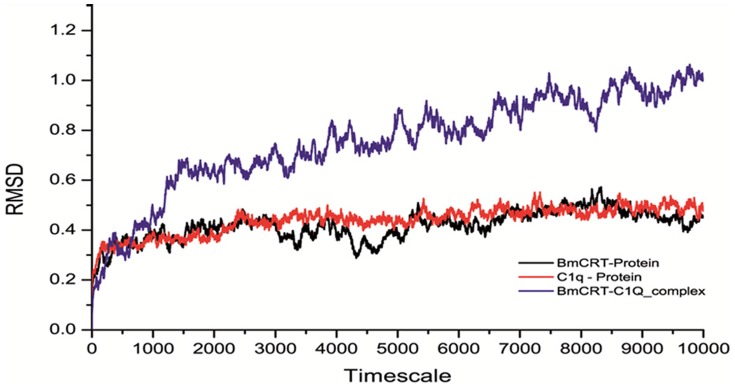
RMSD analysis of apo proteins (BmCRT and HuC1q) and protein-protein complex (BmCRT-HuC1q) with respect to 10 ns (10000ps).

Explicit solvent MDS of BmCRT and C1q protein showed good stability at the simulation point. Overall simulation analysis of both apo proteins simulated for 10 ns and the both protein remained stable throughout the equilibrium condition. The Root Mean Square Deviation (RMSD) of BmCRT and C1q backbone structure with respect to the initial conformation was calculated with respect to function of time period to assess the conformational stability of the protein during the simulations. RMSD of the BmCRT was initially fluctuating from ∼0.3 nm to ∼0.5 nm, but after the 5^th^ ns the protein remains stable till the end of simulation. The RMSD of C1q was more stable and lied in the range of ∼0.35 nm to ∼0.4 nm till the equilibrium state. When comparing the deviations of apo protein with protein-protein complex, there is huge difference seen in complex dynamics (Figure 16). The simulation event showed that protein-protein interaction (PPI) complex was more vigorous in the simulation and due to this the RMSD value of PPI-complex were triggered after 1ns. When comparing the apo proteins, the complex of PPI showed the deviations from ∼0.4 nm to ∼1.0 nm in increasing step by step momentum. In overall comparison of apo and complex protein, the PPI complex was dynamically more active in the solvent condition.

## Discussion

Nematode parasites have large genomes, which are likely to encode a spectrum of products able to block or divert the host immune response. The reports from human as well as animal studies clearly suggest that the impact of nematode infection on the immune response is far more complicated than we would hope and it is obvious that we are in fact rather limited in our knowledge regarding the interactions between these parasites and the host immune system. Several studies have shown that nematodes can influence co-infection [73]–[75] and vaccine [76]–[77] efficacy by modulating hosts immune response. Cathepsin L-like protease [78]–[79], “IL5-like” substrate [80]–[81], GST and CPI-2 [82]–[83] are some E/S products that modulate or evade the host immune mechanism. In the present study we sought to investigate, via theoretical and experimental approaches, the role of BmCRT in host evading capacity as well as explore mechanism of CRT-C1q interaction.

*Brugia malayi* Calreticulin (BmCRT) a Ca^+2^ binding protein is one of such candidate, which was successfully cloned, expressed and functionally characterized for the first time. The native mass (46 kDa) of purified BmCRT indicated its monomeric nature, while CRT from other sources have been reported to be diameric, trimeric, tetra or octameric in nature [42], [84]. The interaction of BmCRT with C1q was studied by modeled as well as purified BmCRT protein. As shown in figure 4, BmCRT interacted in a specific and dose dependent manner with C1q (BmCRT-C1q) under physiological condition. We also carried out pull-down assay to confirm the interaction of both proteins by use of specific antibodies for both proteins (Figure 7). Formation of this complex was found to be responsible for blocking of classical complement system, as confirmed by inhibition of haemolytic activity, mediated by BmCRT (Figure 5). In classical complement system sequential activation of C1q-bound C1r and C1s serine proteases promotes proteolysis of C_4_ to produce C4b which is responsible for complement cascade activation [15], [85]. Thus during the activation of cascade by C1q-C1r_2_C1s_2_ (C1 complex) in the serum due to transfer of a signal from C1q after binding with their targets like IgG/IgM, immune stimulating and membrane attacking complex (MAC, haemolyses of cells) is generated [9], [14], [15]. The compounds interfering with the activation of C1q-C1r_2_C1s_2_ complex caused inhibition of classical complement pathway [86]. Several studies have been conducted elucidating role of human and parasites CRT in interference of C1q activation which leads to inhibition of classical complement pathway [35], [36], [41]–[43]. The present study with BmCRT showed that addition of BmCRT inhibited C1q haemolysis activity in a dose-dependent manner (Figure 5), indicating that BmCRT interacted with C1q, thus inhibiting its ability to activate C1 complex. These experimental evidence showing that BmCRT, by virtue of its capacity to bind and inhibit the function of C1q, played a preponderant role in the defense against this parasite. The studies carried out with three dimensional structure of BmCRT-C1q complex in the current study provided much valuable information on the architecture and chemistry of this protein-protein interaction which indicated inhibition of sequential activation of C1q-bound C1r and C1s serine proteases.

Human CRT was initially characterized as a C1q receptor through its ability to interact with its collagen region (CLF) [29]–[34], Inspite of extensive investigations on HuCRT-C1q interaction, the binding characteristics of human CRT towards C1q is unclear. Many investigators have suggested that HuCRT interacted with collagen like stalk of the C1q [37]–[40], [45], [46], while few studies have shown its interaction through head region of C1q [16], [35], [36]. Recently Paidassai et al., (2011) reported that, GR and CLF domains of C1q recognized CRT with similar affinities (K_D_ values  = 2.6–8.3×10–7 M) and concluded that, in addition to CLF, the GR of C1q also binds to CRT [48]. To address the interaction of BmCRT with C1q we performed a competitive inhibition assay which revealed that the binding of C1q to immobilized BmCRT was strongly inhibited by IgG in a dose dependent manner, while no effect was observed with SAP (shown in figure 8), which strongly suggest the interaction of BmCRT with head region of C1q. To further confirm this, *in silico* studies were conducted and results showed that the binding site is present in the top portion of C1q, between A, B, C chains of C1q. The B chain (Head region) contributed significantly in binding with the BmCRT (Figure 13). Altogether, the *in vitro* and *in silico* results clearly suggested the involvement of head region of C1q in binding with BmCRT and no binding was observed with the collagen-like tail.

Further in order to elucidate the specific binding regions of BmCRT-C1q complex, protein-protein docking studies were also conducted, which clearly showed that amino acids of C1q head region make significant contributions in complex formation. The interacting residue of C1q mainly interacted with negative regions (blue colored region, P domain) of the BmCRT protein, and these negative regions played a vital role in protein-protein complex formation. Due to the active conformational changes occurring in simulation, BmCRT showed more affinity towards the binding with C1q and this was also confirmed by RMSD analysis. The apo proteins of C1q and BmCRT are stable and have moderate movements in the trajectory, but the complex showed more variations and main fluctuations of complex were seen in the tail region of BmCRT. The amino acid residues mainly contributing in the interaction are shown in figure 15. In general, Tyr^107^, Tyr^126^, Thr^139^, Asn^152^ amino acids of N domain and Ala^196^, Asp^199^, Leu^201^, Pro^214^ of P domain of BmCRT formed hydrogen bonds with Gln^C102^, Arg^B109^, Asn^B152^, Tyr^B175^, Asn^B176^, Asp^B201^, Asn^B203^ and Ser^A208^ amino acids of HuC1q. Our analysis revealed that interacting residues are present in both conserved and non conserved regions of N and P domains of BmCRT, which was not reported in previous biochemical and genetic studies conducted on C1q binding with CRT of other organism like human and *H. contortus* [36], [44]. As we assume that peptides and protein are two different entities and binding study with whole protein may showed whole range of interactions. In full length protein may be only some of these sites should have surface oriented [44]. Around 50% of interacting residues involved in H bonding of BmCRT are not found in *T.Cruzi* and nine amino acids are absent in HuCRT but three amino acids Arg^151^, Met^154^ and Ala^196^ are specifically present in *B. malayi*. Both ionic and hydrophobic residues were found to be present at the complex interface, which indicated that both type of interaction may be involved in complex formation and its stability. Previous studies have shown that initiation of the complex formation of C1q with their targets is a highly charge dependent process and further structural changes in the complex are stabilized by non-polar interaction [87]. Most of the BmCRT binding sites on C1q involved Thr^C147^, Asn^B176^, Try^B175^, Arg^B108^, Arg^B109^, Arg^B150^, Asp^B201^, Ser^B204^ and Glu^A209^ residues have been shown to be important for C1q binding to IgG, IgM and CRP etc as summarized in table 3 [21], [22], [88]. These findings are in agreement with our competitive inhibition assay that showed same binding sites on C1q as shown in figure 8A. Thus by inhibiting the function of C1q, BmCRT contributes towards the parasite ability to block complement activation of host.

**Table 3:** Details of amino acids on human C1q holo and apo plane, which involved in C1q-IgG/IgM CRP and C1q-BmCRT complex formation.

	HOLO plane amino acids	APO plane amino acids	Reference
**Human C1q**	Trp^A147^, Lys^A173^, Glu^A209^, Arg^B108^ *, ArgB^109^ *, Arg^B150^, Tyr^B175^, Asn^B176^, Asp^B201^ *, Asp^B104^, His^C101^, His^C167^, Lys^C170^, Asp^C195^	Arg^B108^ *, Arg^B109^ *, Asp^B110^, Gln^B111^, Arg^B114^, His^B117^, Glu^B127^, Arg^B129^, Lys^B132^, Lys^B136^, Glu^B162^, Arg^B163^, Asp^B201^ *	[42], [10]
**HuC1q –IgG/IgM, CRP complex**	Tyr^B175^, Arg^B108^ *, Arg^B109^ *, Lys^C170^, with IgG, IgM and CRP	Arg^B108^ *, Arg^B109^ *, Arg^B114^, His^B117^, Arg^B129^, Arg^B163^ with IgG	[10], [20], [21], [42], [77]
**BmCRT-HuC1q complex**	Glu^A209^, Asp^B104^, Arg^B108^ *, Arg^B109^ *, Arg^B150^, Tyr^B175^, Asn^B176^, Asp^B201^ *, His^C101^, His^C167^, Asp^C195^	Arg^B108^ *, Arg^B109^ *, Asp^B201^ *, Asp^B110^, Gln^B111^	Reported in this manuscript (our findings)

*; Arg^B108^, Arg^B109^ and Asp^B201^ are common in both planes.

Furthermore, studies were conducted to understand the role of Ca^+2^ ions in this interaction. The results with regard to the BmCRT Ca-binding capacity and its role in the inhibition of C1q function as shown in figure 6 indicated that BmCRT inhibited C1q activation both in its holo and apo forms. Most likely, the BmCRT capacity to inhibit C1q function is based mainly on its binding ability directly to C1q. Roumenina et al reported that in the presence of Ca^+2^, the negative end of C1q near the center of mass of the trimer remains unchanged in the holo form but the positive end twists at 67.8° to the quasi-C_3υ_ molecular axis and approaches the B apex (the holo plane) with positively charged residues [55], [10]. The results of BmCRT-C1q docking studies showed that almost all C1q holo plane (B apex) amino acids play major role in complex formation with BmCRT while only few amino acids of apo plane participated in it (Table 3). Thus, in the presence of Ca^+2^ binding affinity of C1q was increased and decreased in absence of Ca^+2^ as confirmed by ELISA. Both DiMoVo server and binding energy studies of BmCRT-C1q complex formation also showed that the presence of Ca^+2^ enhanced the interaction of both proteins.

C1q is normally present in serum as Ca^+2^ bounded form and its interaction with their targets is electrostatic in nature, Ca^+2^ facilitates recognition of negatively charged molecule [10]. This negative field of target molecules by the removal of Ca^+2^ from C1q causes C1q heterotrimer rotation around Arg^B108^-Arg^B109^-Asn^B104^, which could initiate the mechanical stress that gets transmission of activation signal to C1r [55]. Polyanions have been reported to be the ligands of C1q [89] or its inhibitors like B2S [87]. The inhibitory effect of B2S on C1q was enhanced by blocking the release of Ca^+2^ [87]. BmCRT also contains polyanion domains which may promote its interaction towards C1q and help in inhibition of C1q function by preventing the release of Ca^+2^ from it. The docking studies suggested that BmCRT binds near calcium binding region of C1q and conformational change occured in C1q after complex formation with BmCRT, which do not promote release of Ca^+2^ from C1q. Out of four C1q amino acids Tyr^B173^, Asp^B172^, Gln^B179^ and Gln^A177^ involved in binding with Ca^+2^ [10], only Gln^A177^ is free while rest three amino acid are involved in holding of Ca^+2^ ions (Figure S8) after its interaction with BmCRT. Thus, using both experimental and theoretical studies we showed that Ca^+2^ promoted strong interaction of BmCRT with C1q as a result of which Ca^+2^ was not released from C1q after BmCRT-C1q complex formation, so neither proper orientation of C1q takes place nor activation of C1r occurs which finally blocks the activation of classical pathway. These studies may help in understanding the mechanism of deactivation of C1r_2_-C1s_2_ by BmCRT.

BmCRT specific antibodies were used to probe Western blots of *Brugia malayi* extract from different stages of life cycle in order to investigate stage-specific expression and to probe for BmCRT in secretion of adult worm. Protein bands of 46 kDa were detected in the Western blot in all stages of parasite and in secretory product. These results indicated that BmCRT is expressed in different developmental stage of parasites as well as extracted by adult *B. malayi*. This result was further confirmed by C1q binding assay with culture media containing E/S product with BmCRT specific antibody. Culture media with no E/S product showed no interaction with C1q (Figure S9). These results are in agreement with studies of Hewitson et al., on E/S products of *B, malayi* [90]. All these findings indicate that BmCRT is a secretory protein as reported in other nematodes [42], [43], [91]. Further experiments are still in progress to look in depth to investigate other possible roles of BmCRT that influences the host- parasite interactions.

## Conclusion

Finally, based on these observations, it could be proposed that, the 46 kDa secreted BmCRT protein, may contribute modulation of host defense against this parasite. Furthermore we found that C1qB chain apex (IgG, IgM and CRP binding sites on huC1q globular head region) play major role in binding with BmCRT. Thus by interfering C1q binding with IgG/IgM and CRP, BmCRT contributes to parasite ability to block complement activation of host which might be helpful in parasites establishment. To the best of our knowledge this is first report which predicts a complete mechanism of deactivation of C1q-C1r_2_-C1s_2_ by CRT-C1q complex, in which calcium plays significant role. The results presented here have several potential translational medicine aspects, specifically designing the possible inhibitors to inhibit the C1q/CRT interactions and thus *B. malayi* infectivity.

## Supporting Information

Figure S1 **Sequence analysis of both template and input sequence and their secondary structure prediction.**
(TIF)

Figure S2 **Comparative analysis of structure and sequence information's.** (**A**) Modeled protein compared with crystal structure of globular domain of the human CRT (PDB ID = 3POS), showing lack of tail region. (**B**) Sequence similarity between Globular arm domains of Calreticulin (3RG0) and crystal structure of globular domain of the human CRT (3POS).
(TIF)

Figure S3 **Model protein (BmCRT, blue) morphed with 3RG0 (Human CRT, brown).**
(TIF)

Figure S4 **Electrostatic potential surface of Model proteins having more resemblance with template structure.**
(TIF)

Figure S5 **Ramachandran plot of the homology-modeled structure of BmCRT.** The different colored areas indicate “disallowed” (white), “generously allowed” (light yellow), “additional allowed” (yellow), and“most favored” (red) regions.
(TIF)

Figure S6 **Errat quality of Homology modeled structure BmCRT.**
(TIF)

Figure S7 **Crystal Structure of Human C1q with Clock wise and Anti-clock wise rotation.**
(TIF)

Figure S8 **Metal Interactions with before and after protein-protein interactions.**
(TIF)

Figure S9 **Interaction of C1q with BmCRT was observed in adult worm crude and its E/S product.** Microtiter plate was coated with HuC1q (1 µg/ml) in carbonate buffer. After blocking with 5% skimmed milk incubates with rBmCRT (0.5 µg/ml), adult worm crude (25 µg/ml) and E/S products (100 µg/ml). BmCRT specific antibody was used for the detection of BmCRT-C1q interaction in crude and E/S products. No binding was observed in pure culture medium (control). Assay was performed in triplicates. Bar represent the standard deviations of the mean.
(TIF)
