# Supplementary figures and images for: In Silico and In Vitro Studies on the Protein-Protein Interactions between Brugia malayi Immunomodulatory Protein Calreticulin and Human C1q
Source: PLoS One. 2014 Sep 3;9(9):e106413. doi: 10.1371/journal.pone.0106413 (PMC4153637; doi:10.1371/journal.pone.0106413)

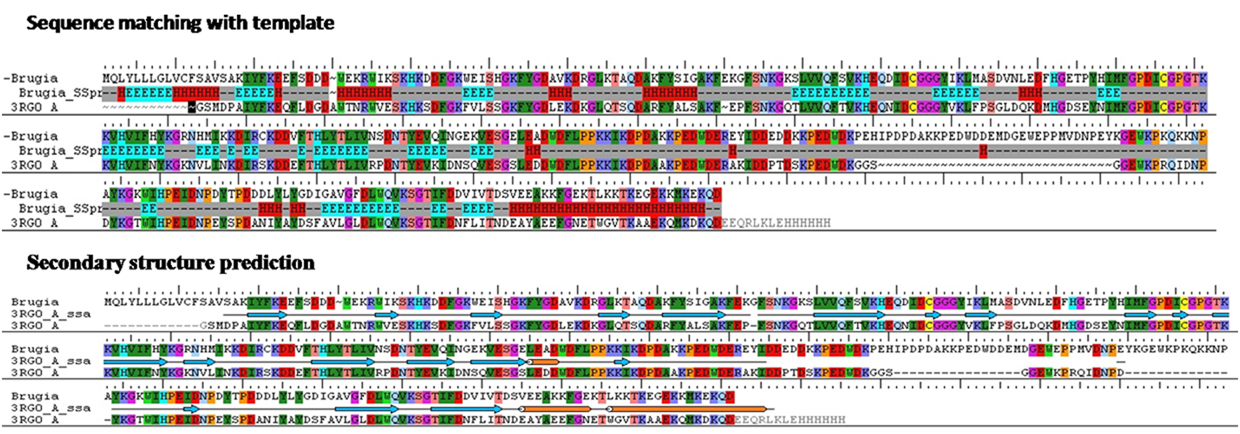

Supplement: Figure S1 — Sequence analysis of both template and input sequence and their secondary structure prediction. (TIF) [file pone.0106413.s001.tif]

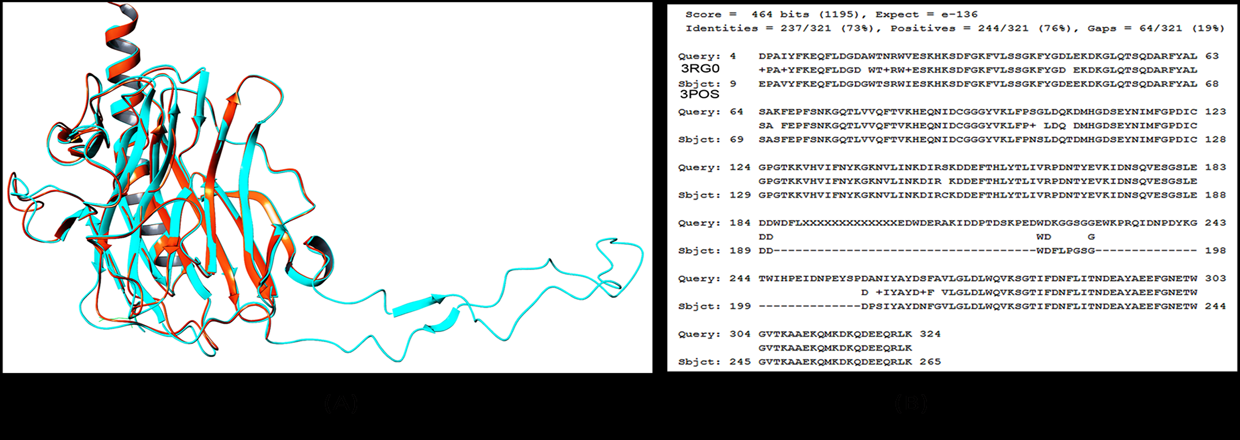

Supplement: Figure S2 — Comparative analysis of structure and sequence information's. (A) Modeled protein compared with crystal structure of globular domain of the human CRT (PDB ID = 3POS), showing lack of tail region. (B) Sequence similarity between Globular arm domains of Calreticulin (3RG0) and crystal structure of globular domain of the human CRT (3POS). (TIF) [file pone.0106413.s002.tif]

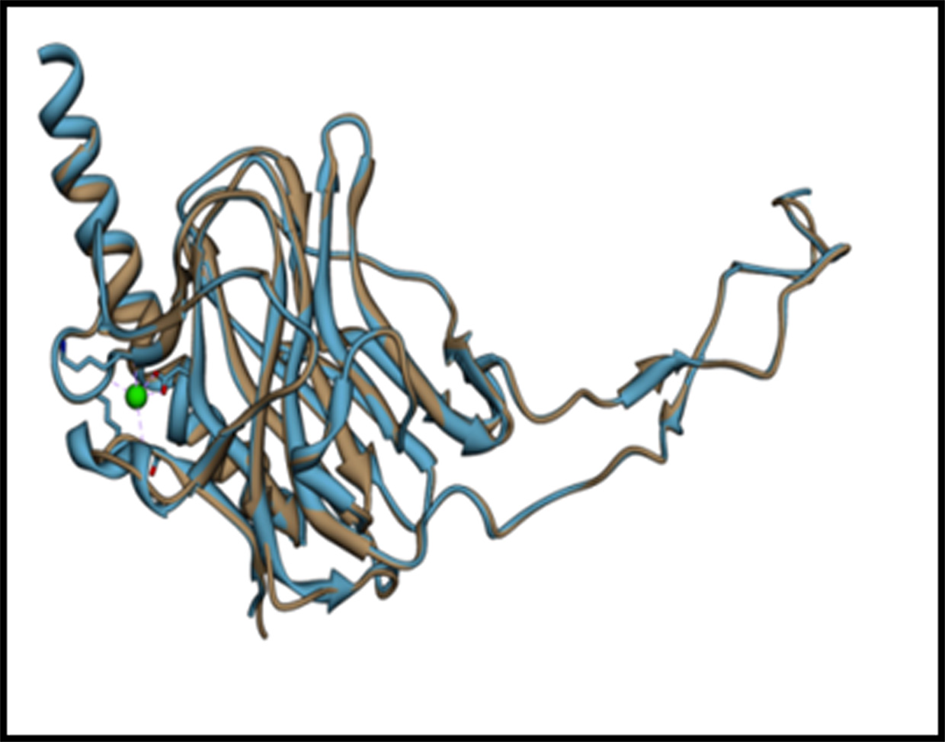

Supplement: Figure S3 — Model protein (BmCRT, blue) morphed with 3RG0 (Human CRT, brown). (TIF) [file pone.0106413.s003.tif]

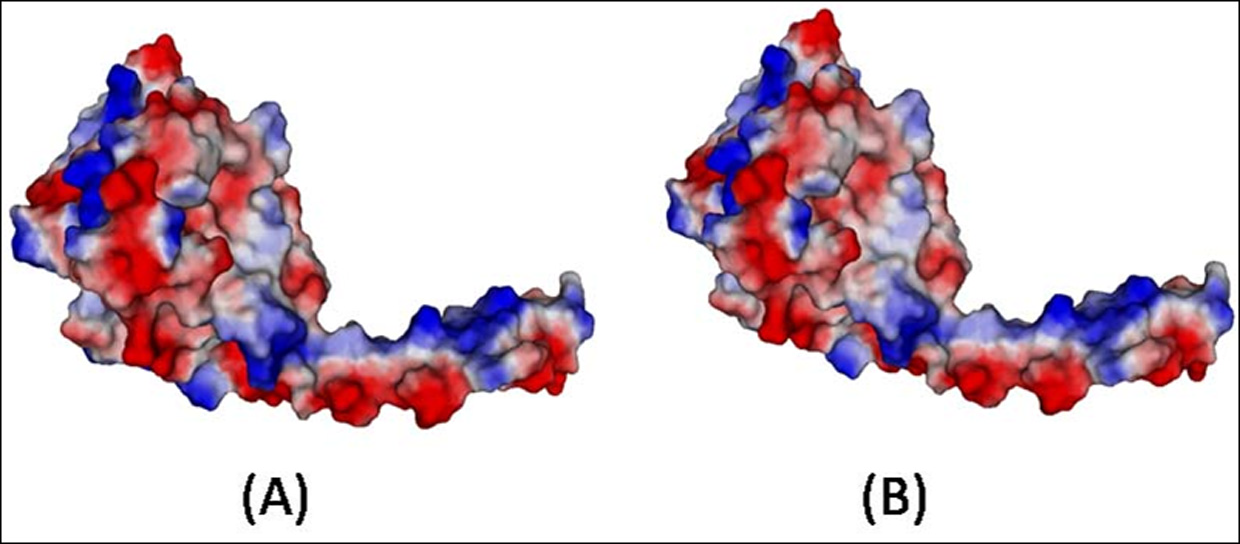

Supplement: Figure S4 — Electrostatic potential surface of Model proteins having more resemblance with template structure. (TIF) [file pone.0106413.s004.tif]

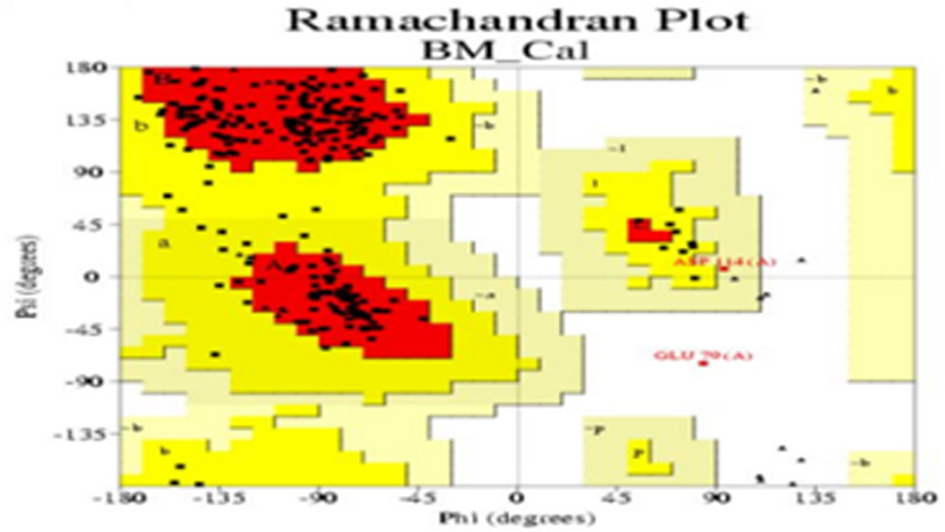

Supplement: Figure S5 — Ramachandran plot of the homology-modeled structure of BmCRT. The different colored areas indicate “disallowed” (white), “generously allowed” (light yellow), “additional allowed” (yellow), and“most favored” (red) regions. (TIF) [file pone.0106413.s005.tif]

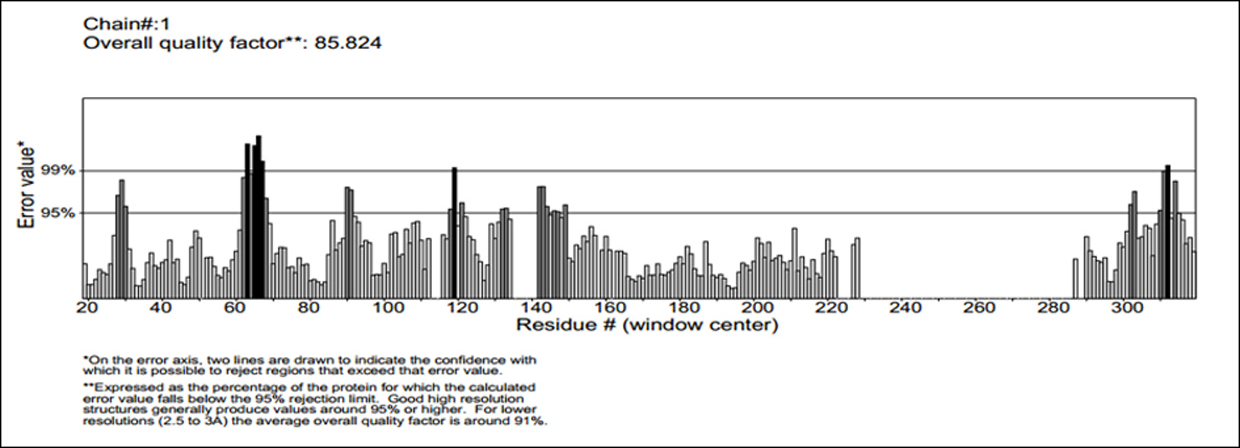

Supplement: Figure S6 — Errat quality of Homology modeled structure BmCRT. (TIF) [file pone.0106413.s006.tif]

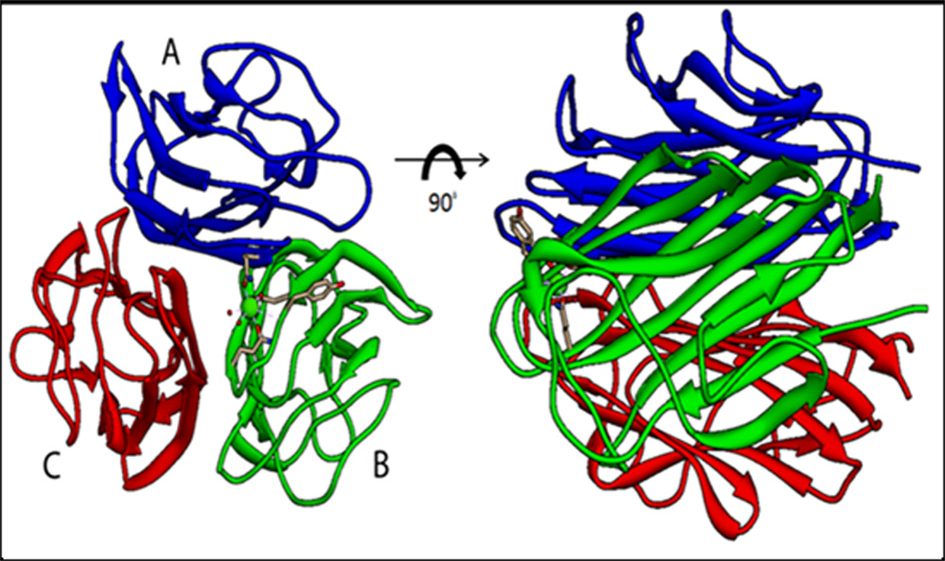

Supplement: Figure S7 — Crystal Structure of Human C1q with Clock wise and Anti-clock wise rotation. (TIF) [file pone.0106413.s007.tif]

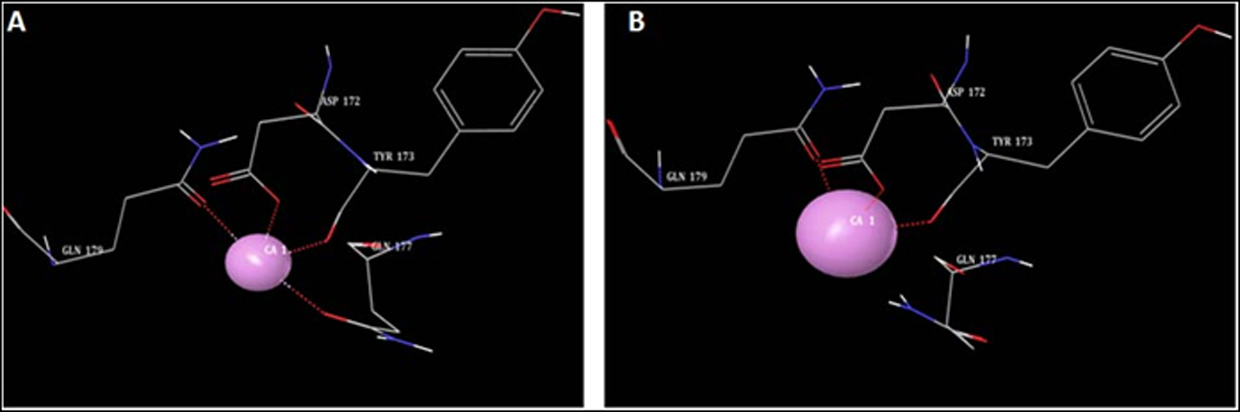

Supplement: Figure S8 — Metal Interactions with before and after protein-protein interactions. (TIF) [file pone.0106413.s008.tif]

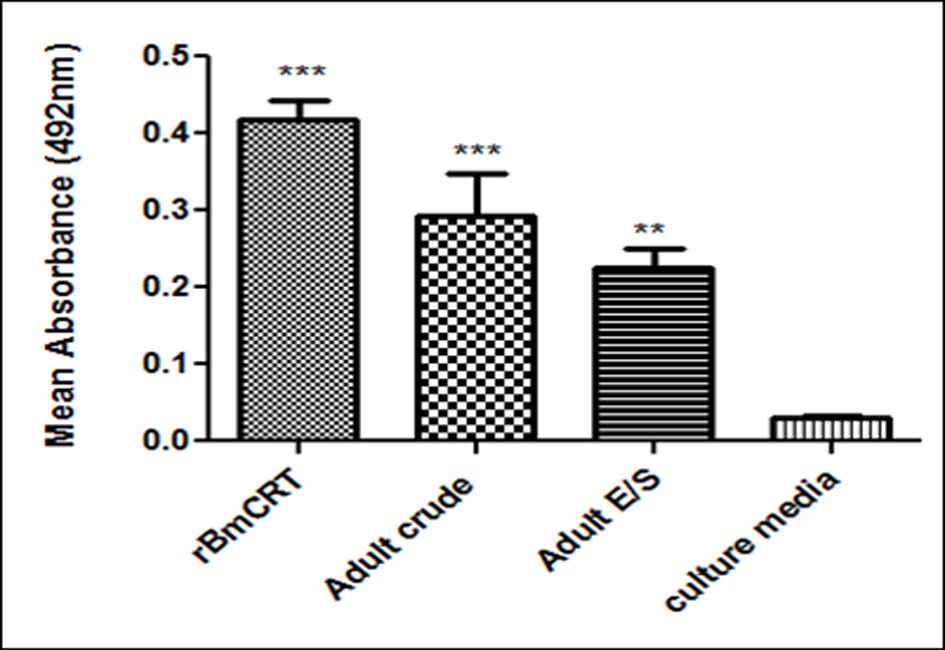

Supplement: Figure S9 — Interaction of C1q with BmCRT was observed in adult worm crude and its E/S product. Microtiter plate was coated with HuC1q (1 µg/ml) in carbonate buffer. After blocking with 5% skimmed milk incubates with rBmCRT (0.5 µg/ml), adult worm crude (25 µg/ml) and E/S products (100 µg/ml). BmCRT specific antibody was used for the detection of BmCRT-C1q interaction in crude and E/S products. No binding was observed in pure culture medium (control). Assay was performed in triplicates. Bar represent the standard deviations of the mean. (TIF) [file pone.0106413.s009.tif]
